# ViE-Take: A Vision-Driven Multi-Modal Dataset for Exploring the Emotional Landscape in Takeover Safety of Autonomous Driving

**DOI:** 10.34133/research.0603

**Published:** 2025-03-14

**Authors:** Yantong Wang, Yu Gu, Tong Quan, Jiaoyun Yang, Mianxiong Dong, Ning An, Fuji Ren

**Affiliations:** ^1^ School of Biomedical Engineering, Anhui Medical University, Hefei, China.; ^2^ Key Laboratory of Knowledge Engineering with Big Data of the Ministry of Education, Hefei University of Technology, Hefei, China.; ^3^ *I*^+^ Lab, School of Computer Science and Engineering, University of Electronic Science and Technology of China, Chengdu, China.; ^4^ Department of Sciences and Informatics, Muroran Institute of Technology, Hokkaido, Japan.

## Abstract

Takeover safety draws increasing attention in the intelligent transportation as the new energy vehicles with cutting-edge autopilot capabilities vigorously blossom on the road. Despite recent studies highlighting the importance of drivers’ emotions in takeover safety, the lack of emotion-aware takeover datasets hinders further investigation, thereby constraining potential applications in this field. To this end, we introduce ViE-Take, the first Vision-driven (Vision is used since it constitutes the most cost-effective and user-friendly solution for commercial driver monitor systems) dataset for exploring the Emotional landscape in Takeovers of autonomous driving. ViE-Take enables a comprehensive exploration of the impact of emotions on drivers’ takeover performance through 3 key attributes: multi-source emotion elicitation, multi-modal driver data collection, and multi-dimensional emotion annotations. To aid the use of ViE-Take, we provide 4 deep models (corresponding to 4 prevalent learning strategies) for predicting 3 different aspects of drivers’ takeover performance (readiness, reaction time, and quality). These models offer benefits for various downstream tasks, such as driver emotion recognition and regulation for automobile manufacturers. Initial analysis and experiments conducted on ViE-Take indicate that (a) emotions have diverse impacts on takeover performance, some of which are counterintuitive; (b) highly expressive social media clips, despite their brevity, prove effective in eliciting emotions (a foundation for emotion regulation); and (c) predicting takeover performance solely through deep learning on vision data not only is feasible but also holds great potential.

## Introduction

Takeover in autonomous driving (L2–L3 automation) refers to the process where a human driver assumes control of the vehicle upon encountering a situation that it cannot handle or exceeds its designed capabilities [1,2]. It often implies a tricky situation or an immediate danger, such as pedestrian avoidance [3], where a small error (like a missed warning) could result in a big disaster (like a fatal car crash). As a result, takeover safety draws increasing attention as the pioneering autopilot vehicles face more and more challenges in real-world situations nowadays.

The key to takeover safety is the driver’s performance, which is affected by various driving contexts [4], like the driver’s physical state [5–7], the external driving environment [8–10], types of non-driving-related tasks (NDRTs) [6,11,12], and human–machine interface [13,14]. One possible way to leverage these factors is to set up offline regulations for precaution. But precautions will not help vehicles to foresee whether a particular driver can handle a particular takeover event well. Therefore, online prediction of drivers’ performance in takeovers via computational models attracts much attention currently. In general, they leveraged driver monitoring systems [15] (DMSs) to collect drivers’ physical data (e.g., eye movements and head pose [16–18]) with various driving-related contexts (e.g., vehicle [18], traffic [19], and weather [17]) to predict different aspects of takeover performance such as readiness [2,19] and reaction time [17].

Recently, emotion [20–23], another major context affecting human behaviors and performance [24,25], is being examined for its impact on takeover performance with inspiring observations reported [26]. The intuition behind is that takeover is a complex task involving attention, information perception, real-time judgment, and decision execution [27], all affected by emotions [20,24,28]. However, despite the discovery of emotion’s important on takeover performance, the absence of public emotion-involving takeover datasets limits its further exploration and potential applications in practical driving scenarios. “Related research” section compares existing public datasets on takeover performance understanding and highlights the absence of the emotional factor in current datasets.

**Fig. 1.**
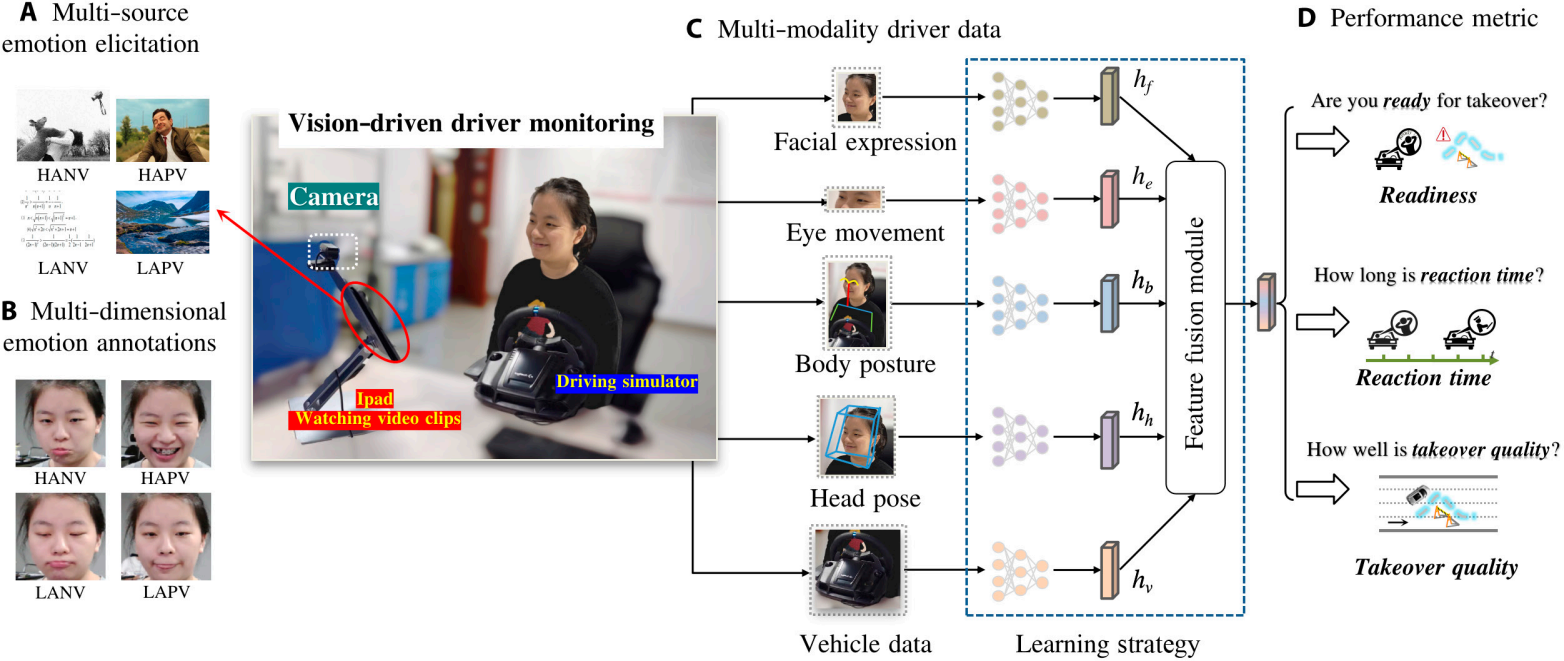
Overview of the proposed ViE-Take dataset. (A) Multi-source emotion elicitation. (B) Multi-dimensional emotion annotations. (C) Multi-modality driver data, including facial expression, eye movement, body posture, head pose, and vehicle data. (D) Three performance metrics, i.e., readiness, reaction time, and quality.

To this end, we introduce ViE-Take, the first Vision-driven dataset for charting the Emotional landscape in Takeovers of autonomous driving. As shown in Fig. 1, ViE-Take offers 3 distinctive characteristics to better understand the role of emotions in takeovers: (a) multi-source emotion elicitation: carefully selected clips from social media and movies to elicit diverse emotions of drivers; (b) multi-modal driver data: a front camera monitoring the driver’ face, eyes, head, and upper body; and (c) multi-dimensional emotion annotation: an information-rich and computation-friendly 2-dimensional (2D) model [i.e., valence-arousal (VA) model] for labeling driver emotions instead of the traditional categorical model (explained later in the “Emotion modeling” section).

To aid the use of ViE-Take, we provide 4 elementary deep models as benchmarks for takeover performance prediction, corresponding to 4 learning strategies (2D, 2D + Timing, 2D + Timing + Attention, and 3D, explained later in the “Benchmarks for takeover performance prediction” section). To facilitate holistic understandings of emotion in the takeover, we also present preliminary analysis on various issues, e.g., emotion elicitation in driving and correlation between emotions and takeover performance.

In summary, our work makes the following main contributions:

• The first public dataset (to the best of our knowledge) designed to investigate how emotion qualitatively and quantitatively affects different aspects of takeover performance (i.e., readiness, reaction time, and quality).

• Rich emotion stimulus (i.e., clips collected from social media and movies) with manual labels, which is proved to be capable of eliciting diverse emotions and thus can serve as the foundation for a wide range of emotion-related studies in emotion recognition and regulation.

• Four pretrained deep models with full codes and parameters for takeover performance prediction, which can benefit various downstream tasks (like emotion recognition and regulation) of automobile manufacturers (usually favoring the same vision-based DMS) for enhancing their autonomous driving systems.

The remaining sections are organized in the following manner. The “Results” section describes the ViE-Take dataset and presents benchmark networks for takeover performance prediction. The “Discussion” section analyzes the correlation between emotions and takeover performance, explores open issues such as challenges in emotion stimulation, and summarizes our work. In the “Methods” section, we review the related work and describe the methods used for building the datasets. Additionally, the “Acknowledgements” and “Data Availability” sections are included to acknowledge contributions and provide information regarding the availability of the data.

## Results

Despite the significance of emotion in takeover performance, there currently does not exist an emotion-involved public dataset to facilitate a holistic understanding of emotions in takeover performance. More importantly, such a dataset is valuable in aiding the design and pretraining of an online prediction system for takeover performance. In light of this, we present ViE-Take, the first Vision-driven dataset for exploring the Emotional landscape in Takeover safety.

### Dataset

ViE-Take includes multi-source emotion elicitation, multi-modal driver data, and multi-dimensional annotations of both emotion and takeover performance. These distinctive aspects enable a comprehensive understanding of how emotions influence takeover behavior in autonomous driving.

#### Dataset content

As shown in Table 1, ViE-Take includes data from 21 participants (12 males and 9 females). Each participant completed 3 takeover events in each emotional quadrant, resulting in 3 (takeovers) × 21 (participants) = 63 takeover records. In total, 63 (takeovers per quadrant) ×4 (quadrants) = 252 takeover records were collected. The driving scenario involved participants navigating a straight line while performing the task of avoiding obstacles.

**Table 1.** Dataset content. C, channels; F, frames; H, height; W, width; P, position; s, second.

No. of participants	21 (12 males and 9 females)
Driving scenario	Straight line
Driving tasks	Avoiding the obstacles
Emotion elicitation	Watching video–audio clips
No. of TOR events	252 records (3 TORs × 21 participants × 4 quadrants)
	Recorded data	
Driver driving video	- Facial expression stream
	RGB 3C × 30F/s ×10s×256H×256W
	- Head pose stream
	Keypoint30F/s ×10s× 6P
	- Eye movement stream
	Keypoint30F/s ×10s× 288P
	- Body posture stream
	Keypoint30F/s ×10s×12P ×2coordinates
Vehicle data	- Velocity 30F/s × 10s
	- Throttle pedal30F/s × 10s
	- Brake pedal30F/s × 10s
	- Steer wheel30F/s × 10s
Data labeling	
Emotional state	- Valence (scale 1-9)
	- Arousal (scale 1–9)
Takeover performance	
Subjective	Readiness (scale 0–1)
	Reaction time (scale 0–2)
	Takeover quality (scale 0–4)
Objective	- Acceleration - Jerk
	- Brake - Steer - ${\mathrm{TTC}}_{\min}$

For emotion elicitation, we chose audiovisual clips (see the “Multi-source emotion stimulus selection” section), which are crucial for studying the correlation between drivers’ emotional states and takeover performance. We collected multi-modal driver data, including the driver’s facial expressions, head pose, eye movements, and body posture (see the “Multi-modal driver dataset collection” section). Additionally, vehicle data such as velocity, throttle pedal, brake pedal, and steering wheel were recorded. The data were labeled with both emotional states (valence and arousal, see the “Emotion modeling” section) and takeover performance indicators (see the “Takeover performance indicators” section).

#### Emotion modeling

**Fig. 2.**
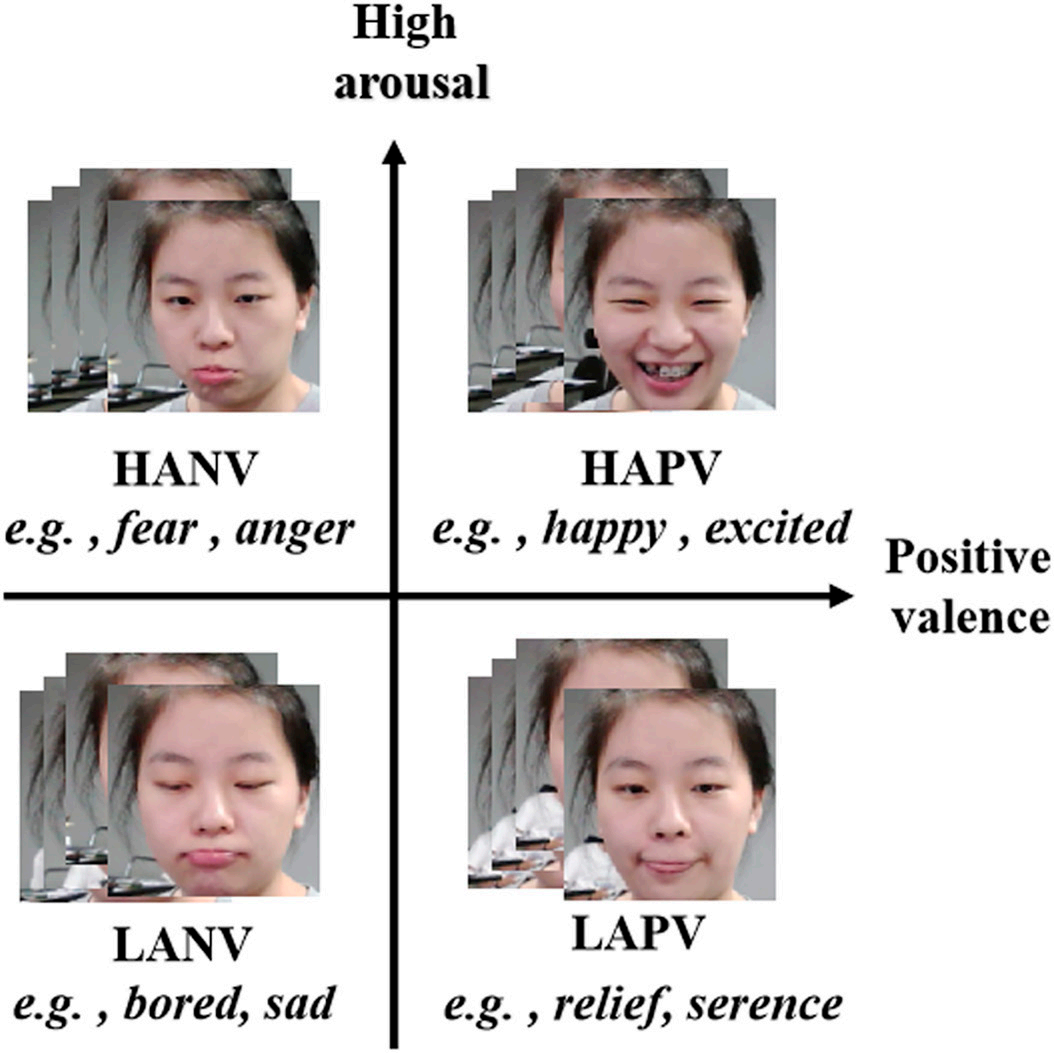
Emotion modeling: A joint example of categorical and continuous emotion annotations.

As shown in Fig. 2, 2 emotion representations have been used frequently in computation [29]. One is the categorical model, where emotions are discrete and fundamentally different constructs like anger, fear, and happiness. The other is the dimensional model where emotions can be characterized dimensionally in groupings, e.g., the well-recognized VA model. Here, valence means the degree of negativity or positivity of a stimulus, whereas arousal indicates its level of calmness or excitement [30]. We choose the latter since it allows computation and comparison between emotions due to the use of numerical vectors for emotion representation.

#### Takeover performance indicators

Takeover performance is a general term that includes various indicators measuring driver behaviors during a takeover [10,31]. Previous studies have employed a range of metrics, covering aspects such as readiness, reaction time, takeover quality, and vehicle-related data. Here, we select 3 prevalent indicators to systematically evaluate takeover performance, i.e., readiness, reaction time, and quality, as outlined in Table 2.

**Table 2.** Details on takeover performance indicators

Readiness: Drivers’ readiness to take over the vehicle	
Subjective	
- Ready	Prepare for takeover
- Not ready	Complete unpreparedness
Reaction time: Measuring the drivers’ time delay	
Subjective	
- Short	( *0* s, μ - σ ]
- Medium	( μ - σ, μ + σ ]
- Long	( μ + σ, ∞ ]
*Follows a normal distribution with* μ*(mean) and* σ*(variance)*	
Objective	Recording the drivers’ time delay (from perceiving to action)
Takeover quality: Evaluating the driver’s control proficiency	
Subjective
Level_1	Complete loss of control
	(e.g., collisions)
Level_2	Endangerments of oneself or others
	(e.g., near misses)
Level_3	Occurrence of driving errors
	(e.g., late or insufficient braking)
Level_4	Imprecisions of vehicle control (e.g., imprecise lane keeping)
Level_5	Perfect performance (e.g., absence of imprecisions)
Objective	
Acceleration	Smoother and safer reactions
Jerk	The derivative of acceleration
Brake	The angle of brakes during avoidance
Steer	The angle of steering wheel
${\mathrm{TTC}}_{\min}$	Minimum time to collision

##### Readiness

Takeover readiness refers to the degree to which participants were prepared to assume control of the vehicle upon receiving a takeover request (TOR) [32]. We use the term “Not Ready” to denote complete unpreparedness for the takeover, while any other response is interpreted as “Ready” for the takeover.

##### Reaction time

Reaction time is a widely used objective metric for assessing takeover performance [33]. One popular definition is the duration it takes the driver to redirect their focus to the road after being distracted by a visual NDRT [34]. But it is difficult to precisely measure in reality since looking on the road is difficult to capture.

In this study, we specifically focus on operational reaction time, which calculates the human time delay from the perception of a TOR to the execution of an action. To this end, takeover reaction time refers to the period from the TOR reminder to the moment the steering wheel button is pressed for control transition.

Based on our experiments, we found that operational reaction time follows a normal distribution. Hence, we computed both the mean and standard deviation to systematically characterize the distribution of reaction time. Building on this observation, we categorized operational reaction time into 3 categories, i.e., “Short”, “Medium”, and “Long”, as detailed in Table 2.

##### Quality

Takeover quality indicators encompass both objective and subjective measures. Objective indicators evaluate vehicle data, such as acceleration, where smaller values indicate smoother and safer reactions to TORs. Jerk, the derivative of acceleration, is used to assess shift quality, ride comfort, and driving aggressiveness. Additionally, minimum time to collision (${\mathrm{TTC}}_{\min}$) acts as a time-oriented safety measure to identify the risk of rear-end collisions. Subjective evaluation indicators, as outlined in ISO/TR 21959-1 [35], represent recorded values during driving. These indicators assess a driver’s control proficiency during transitions and consolidate various aspects of the takeover situation into a single measure of overall driving quality.

### Benchmarks for takeover performance prediction

This section introduces 4 elementary deep models as benchmarks for takeover performance prediction, representing popular learning strategies like 2D and 3D, and all consisting of mainstreaming and easy-access learning modules like ResNet18 [36]. These benchmarks not only demonstrate the feasibility of leveraging deep learning in performance prediction but also suggest the possibility of potential prediction improvements with cutting-edge learning techniques like vision transformer [37].

#### Benchmark rationale

The choice of benchmark methods is crucial in assessing the performance of takeover performance prediction models. We aim to include representative deep learning methods to establish a strong baseline answering the following questions: (a) whether it is feasible to predict takeover performance using only vision data; (b) whether emotions impact the prediction results.

#### Prediction methods

As shown in Table 3, our vision data are essentially videos, which are usually handled by the following learning strategies:

**Table 3.** Prediction result of the 4 elementary deep models. MLP, multilayer perception; LSTM, long short-term memory; DNN, deep-learning neural network.

Method	Backbone (learning strategy)					Self-reported emotion quadrant	Readiness		Reaction time		Quality	
	Facial expression	Eye movement	Head pose	Body gesture	Vehicle data		ACC	F1	ACC	F1	ACC	F1
2D	ResNet18 [36]	MLP	MLP	MLP	MLP	HANV	72.34	73.59	48.94	46.44	53.19	56.09
						HAPV	88.37	93.04	46.51	46.37	41.86	47.03
						LAPV	60.86	64.03	52.17	52.87	36.23	37.86
						LANV	58.54	63.66	70.73	67.54	39.02	40.43
						Average	67.46	65.19	54.76	54.68	42.06	40.86
2D+ Timing	ResNet18+ LSTM	LSTM	LSTM	LSTM	LSTM	HANV	72.34	69.57	55.32	54.75	42.55	42.97
						HAPV	90.69	93.09	60.47	58.44	46.51	49.16
						LAPV	75.36	75.16	53.62	54.05	40.58	39.36
						LANV	75.61	71.80	56.1	61.55	31.70	29.16
						Average	77.38	78.54	56.35	56.36	39.68	40.70
2D+ Timing+ Attention	ResNet18+ LSTM +Attention	LSTM + Attention	LSTM + Attention	LSTM + Attention	LSTM+ Attention	HANV	78.72	72.20	70.21	68.95	44.68	42.16
						HAPV	90.69	94.14	60.47	57.56	55.81	53.45
						LAPV	76.81	74.41	63.76	62.18	49.28	46.41
						LANV	70.73	67.53	70.73	68.57	39.02	32.19
						Average	77.38	79.76	65.07	64.95	45.25	48.59
2D+ Timing+ Attention	ResNet18+ Transformer[94]	Transformer	Transformer	Transformer	Transformer	HANV	80.85	70.97	51.06	51.08	65.96	63.07
						HAPV	97.67	68.53	60.47	58.67	34.88	30.19
						LAPV	76.81	74.37	57.97	55.48	47.83	40.83
						LANV	70.73	68.69	41.46	36.92	41.46	36.75
						Average	79.78	70.86	52.38	49.91	48.05	41.65
2D+ Timing+ Attention	ResNet18+ Reformer[95]	Reformer	Reformer	Reformer	Reformer	HANV	79.76	71.34	51.09	51.18	62.91	60.07
						HAPV	94.23	92.04	59.30	58.27	38.38	31.19
						LAPV	75.29	73.82	56.28	55.36	42.74	41.01
						LANV	71.45	69.75	50.27	54.61	38.46	38.75
						Average	77.02	74.53	57.16	56.64	44.49	43.90
2D+ Timing+ Attention	ResNet18+ Autoformer [96]	Autoformer	Autoformer	Autoformer	Autoformer	HANV	70.32	71.27	51.06	51.08	62.38	56.47
						HAPV	87.27	80.39	58.84	49.67	38.77	31.72
						LAPV	75.34	72.45	57.97	52.24	44.95	38.61
						LANV	70.71	65.91	45.24	44.92	41.46	40.01
						Average	73.83	73.36	46.03	43.47	45.23	40.20
3D	ShuffelNet V2 [38]	DNN	DNN	DNN	DNN	HANV	79.55	80.16	54.55	55.56	34.09	29.51
						HAPV	83.72	87.78	51.16	49.41	44.18	41.70
						LAPV	64.61	66.85	47.69	46.03	41.54	34.09
						LANV	71.05	63.55	50.00	44.64	39.47	38.16
						Average	75.00	73.62	49.17	47.02	38.75	35.99

1. 2D: A video is treated as separate 2D images (30 images per second). For the facial expression on each image, we leverage the well-recognized and widely used ResNet18 [36] for feature extraction. For other modality data, we apply multilayer perceptrons (MLPs) with ReLU activation as encoders.

2. 2D + Timing: A video is treated as sequential 2D images. Therefore, we leverage the long short-term memory (LSTM) backbone (batch size: 300) to capture the temporal correlations between images for all modality data.

3. 2D + Timing + Attention: A global attention is applied to the concatenated features output by the 2D + Timing strategy (as shown in learning strategy part of Fig. 1; $h_{f}$, $h_{e}$, $h_{b}$, $h_{h}$, and $h_{v}$ represent features of face, eye movement, body posture, head pose, and vehicle data, respectively) in order to capture the spatial correlations between modalities.

4. 3D: The 3D network structures directly model hierarchical representations of an image. We tried popular models like ShuffleNet V2 [38] for takeover performance predicition.

The above deep learning methods are trained in the same hardware and software environment, which consists of an NVIDIA GeForce RTX 4090 GPU on Ubuntu 18.04.6 LTS with CUDA 12.1 for GPU acceleration. The GeForce RTX 4090, known for high performance, enabled efficient parallel processing that is crucial in training. The Adam optimizer [39] is utilized with a learning rate of 0.005. Moreover, image augmentations such as random horizontal flips, random cropping, and random rotation were implemented to effectively augment the training dataset.

#### Evaluation metrics

We utilize 2 metrics, namely, *accuracy* and *weighted_F1_score*, to assess the system’s performance.

$$\text{Accuracy}=\frac{TP+TN}{TP+FP+TN+FN}$$ (1)

$$\text{Weighted\_F}1\_\text{Score}=\sum 2\times \frac{\text{Precision}\times \text{Recall}}{\text{Precision}+\text{Recall}}\times W_{i}$$ (2)

Here, TP, TN, FP, and FN represent true positive, true negative, false positive, and false negative, respectively. Precision $\left(\text{Precision}=\frac{TP}{TP+FP}\right)$ signifies the ratio of true positive examples in the prediction outcome, while Recall $\left(\text{Recall}=\frac{TP}{TP+FN}\right)$ indicates the proportion of correctly predicted positive instances. $W_{i}$ denotes the weight of the predicted category, where *i* denotes the number of categories. Higher *accuracy* and *weighted_F1_score* reflect superior network performance.

#### Prediction results and analysis

Table 3 compares the prediction results in *accuracy* and *weighted_F1_score* of the 4 learning strategies. Moreover, we report their performance in each emotional quadrant to understand how emotion affects the prediction. We conclude the experimental results as follows:

Q1. Whether it is feasible to predict takeover performance using only vision data?

Ans. Yes, in short. The best prediction accuracy for the takeover readiness, reaction, and quality is 79.78%, 65.07%, and 48.05%, respectively. They are all achieved by the “2D + Timing + Attention” strategy. Considering that predicting takeover quality is a 5-class classification problem (very hard in general), we can safely conclude that it is possible to predict takeover performance with only vision data.

Q2. Whether emotions impact the prediction results?

Ans. Yes, in general. For example, the highest prediction results are almost achieved all in the HAPV (high arousal and positive valence) quadrant (emotions like happy and excited), for all 4 learning strategies. We think that is because emotions in HAPV usually are the most expressive (e.g., facial expressions and eye movements). In contrast, the lowest prediction results tend to appear at the LA (low arousal) end.

Note that here we only address basic questions in takeover performance prediction. There still exists many open questions for further exploration. Here are some examples:

1. The prediction results show steady improvements as we escalate the learning strategies. Whether the state-of-the-art vision techniques like the Transformer Encoder (TransE) could further boost the prediction performance?

2. Emotions appear to affect the prediction results. Whether this observation is related to the fact that facial images have the largest amount of data compared to other modalities?

3. We leverage facial expressions images directly. But we think that facial expressions should be sufficiently coded (like in action units or an automated arousal and valence detector) to better understand how emotion matters in takeover.

## Discussion

### Correlation between emotion and takeover performance

To investigate the impact of emotions on takeover performance, we conducted a correlation analysis between valence/arousal and the dependent variables of takeover performance (both subjective and objective indicators, detailed in Table 2). Please note that participants’ self-reported emotions serve as the ground truth for further analysis.

#### Emotion and takeover performance (subjective indicators)

**Fig. 3.**
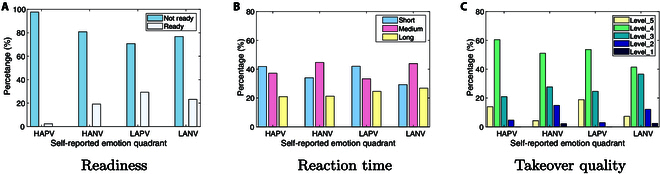
Distribution of measures (subjective indicators) among readiness (A), reaction time (B), and takeover quality (C) under 4 emotional quadrants.

In Fig. 3, we illustrate the distribution of takeover performance measures, specifically subjective indicators, across takeover readiness (Fig. 3A), reaction time (Fig. 3B), and takeover quality (Fig. 3C) within different quadrants.

##### Readiness

Figure 3A illustrates the readiness proportions in different emotional states. It is interesting to see that under high arousal, participants tend to be in a “Not ready” state, while those in low arousal are “Ready” for takeover. Particularly in the LAPV (low arousal positive valence), the “Ready” state is most prevalent.

##### Reaction time

In Fig. 3B, reaction time proportions are depicted across various emotional states. Under positive valence, participants show a higher proportion of “short” reaction time, particularly in LAPV at 42.03%. In contrast, “Long” reaction times are often observed in negative valence; for example, HANV exhibits 44.68% “Medium” reaction time, while LANV has 26.83% “Long” reaction time.

##### Takeover quality

Figure 3C illustrates the takeover quality proportions in different emotional states. In LAPV state, participants tend to have good takeover quality. In contrast, drivers have the worst takeover quality in HANV and LANV states, even resulting in collisions (i.e., “Level_1” quality). Considering the previous analysis, this is likely due to the fact that participants experience longer reaction time in negative valence, which is insufficient for a smooth transition into the takeover state, thereby resulting in poorer quality.

#### Emotion and takeover performance (objective indicators)

We employed Spearman tests to separately assess the monotonic correlations between valence and arousal with takeover performance.

For valence, we found a statistically significant difference in its impact on ${\mathrm{TTC}}_{\min}$ (t=−0.24,p<0.005). This variation may be associated with the positive influence of emotions on driving safety. Meanwhile, no other significant effects were observed.

Regarding arousal, we observed statistically significant differences in its effects on steering angle (t=−0.18,P<0.003) and braking (t=−0.15,P<0.02). This suggests that higher arousal levels may result in smaller steering and braking angles.

### Emotion stimulation

In the material selection phase (as the “Multi-source emotion stimulus selection” section), we constructed an emotion stimulus database using movie clips and short videos from social media, emphasizing the efficacy of stimuli in inducing target emotions.

#### Stimulation challenges

We faced the following 3 challenges in preparing emotion elicitation materials using the traditional move clips:

Inefficient movie editing: Editing movie scenes demands substantial manpower and time. As nonprofessional movie editors, individual interpretations of emotions may hinder the effectiveness in stimulating the overall target emotions.

Quick emotion stimulation: The complexities of character backgrounds, story development, and plotlines in movies can impede participants’ comprehension of emotional evolution, influencing stimulus effectiveness.

Prolonged emotion retention: While movies often demand immersive experiences, their effectiveness in stimulating high-arousal emotions is limited.

In essence, due to the advantage of material availability, short videos are more advantageous than movie segments in emotion research. First, short videos, with labeled attributes, mitigate the need for additional editing, streamlining the collection process for greater convenience. Second, short videos, by sidestepping intricate character relationships and prioritizing emotional expression, efficiently elicit emotions. Moreover, short videos are designed to swiftly elicit users’ emotional states within a brief time frame, aligning more with our experiment’s requirement for short-term stimulation. Last but not least, short videos excel in maintaining user emotions, even in high-arousal scenarios, making them effective for experimental requirements.

#### Types of stimulation materials

In this part, we will analyze interview data to determine which stimulus type, between short videos and movie segments, proves more effective in emotion stimulation, utilizing participants’ ratings as key metrics.

**Fig. 4.**
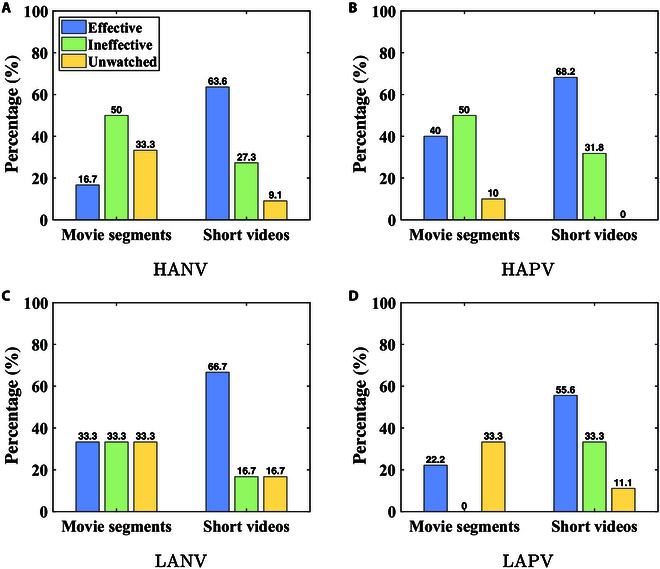
Stimulus material analysis. (A) HANV. (B) HAPV. (C) LANV. (D) LAPV.

During the experiment, participants were asked to score each viewed video on a scale of 0 to 1, where 1 indicates effective target emotion stimulation and 0 indicates ineffective target emotion stimulation. Each video could receive a maximum of 21 points (21 participants), and in some cases, it might receive no points at all. Subsequently, we calculated the average score for each video, categorizing scores from 0 to 0.5 as ineffective and 0.5 to 1 as effective. Finally, we compiled the ratio of effective and ineffective videos, along with the percentage of videos that were not watched. The results for each quadrant are illustrated in Fig. 4.

We find that participants predominantly perceive short videos as more effective than movie segments, notably in the HANV quadrant, with a significant 46.9% difference in effectiveness between the 2 stimulus types. Intriguingly, a considerable number of movie segments remained unexplored during the experiment, particularly in the HAPV quadrant.

Therefore, we can conclude that participants prefer to choose short videos than movie segments for emotion stimulation. This spurred a more in-depth inquiry to unveil the root causes. Through proactive interview with participants, we have distilled the following key conclusions.

#### Participant interview response

We interviewed participants to understand why they perceive the current video–audio stimulus effective. Key findings emphasize the video’s ease of comprehension and level of empathy. All interviews are documented, summarizing participants’ impressions of emotional stimuli (movie segments and short videos) based on ease of understanding and empathy.

In relation to the HAPV quadrant, most stimuli are actually derived from short videos. Both movie segments and short videos all exhibit comparable levels of stimulus effectiveness and evoke significant empathy. Moreover, they are relatively straightforward to comprehend. However, short videos often feature fresher, more relevant content, being closely tied to real-time events and updated rapidly. Therefore, they tend to be more effective, as people naturally gravitate toward novel experiences.

P17 expressed a preference for short videos over movies especially in HANV quadrant. The main reason is that familiar movie segments (i.e., female lead dying after falling from a great height in *Spider-Man 2*), especially those with known endings, lacked significant emotional impact. Conversely, unfamiliar movie segments led to confusion about character relationships and story development, emphasizing plot clarity over emotional stimulation.

In the context of the LANV quadrant, we observed that the more one understands the content of video–audio clips, the less likely it is to evoke the target emotion. This is because the dominant emotion in this quadrant is boredom. We intentionally selected monotonous and dull mathematical analysis lesson videos to stimulate participants’ feelings of boredom and a subdued state.

For the LAPV quadrant, aiming to elicit a state of relief for users, we opted for visually appealing landscapes. We selected the ScreenPeace screensaver featuring city scenes, such as the scenic beauty of European towns and music videos by Jay Chou. These choices are intended to stimulate a relaxed emotional response from users.

### Response emotion (self-reported) versus stimuli emotion (target)

In our experiment, participants utilized self-reported scales [self-assessment manikin (SAM)] to assess their emotions after each TOR. The emotional state (where arousal or valence equals 0) is labeled as marginal. Additionally, the target emotion is determined based on the specific quadrant participants aim to elicit.

#### Inducing rates between self-reported and target emotions

**Fig. 5.**
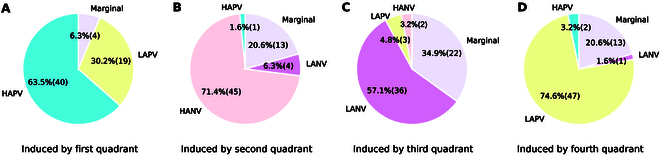
Percentage of participants having watched each quadrant of emotion. (A) Induced by first quadrant. (B) Induced by second quadrant. (C) Induced by third quadrant. (D) Induced by fourth quadrant.

Referring to Fig. 5, we observe inducing rates of 63.5%, 71.4%, 57.1%, and 74.6% in the 4 quadrants. These rates surpass the 50% threshold, confirming the effectiveness of our selected emotional stimuli material.

While our emotion elicitation method is easy to use and efficient, there may be slight differences between target and self-reported emotions. Given the inherently personalized nature of emotions, unraveling these intrinsic relationships is crucial. Subsequent sections will delve into interpreting target emotions with a focus on gender differences.

#### Gender-based variations

As highlighted in Table 4, gender significantly shapes emotional responses (self-reported emotion). The data underscore diverse patterns in how individuals of different genders respond to emotional stimuli (target emotion).

**Table 4.** Gender-based distribution of participants’ responses to emotional stimuli. H, high; L, low; P, positive; N, negative; A, arousal; V, valence; Mar., marginal.

Stimuli (target emotion)	Gender	Response (self-reported emotion)				
		HAPV	HANV	LANV	LAPV	Mar.
HAPV	Female	85.2%	0.0%	0.0%	14.8%	0.0%
	Male	47.2%	0.0%	0.0%	41.7%	11.1%
	All	63.5%	0.0%	30.2%	0.0%	6.4%
HANV	Female	0.0%	77.8%	0.0%	0.0%	22.2%
	Male	2.8%	66.7%	11.1%	0.0%	19.4%
	All	1.6%	71.4%	6.4%	0.0%	20.6%
LANV	Female	0.0%	7.4%	55.6%	0.0%	37.0%
	Male	0.0%	0.0%	58.3%	8.3%	33.3%
	All	0.0%	3.2%	57.2%	4.8%	34.9%
LAPV	Female	7.4%	0.0%	3.7%	59.3%	29.6%
	Male	0.0%	0.0%	0.0%	86.1%	13.9%
	All	3.2%	0.0%	1.6%	74.6%	20.6%

For positive valence, a significant gender difference is observed. In the HAPV quadrant, 85.2% of female participants reported effective emotional stimulation, surpassing male participants by 47.2%. Conversely, for the LAPV quadrant, 59.3% of female participants reached effectiveness, representing a 26.8% decrease compared to male participants. Moreover, differences in arousal levels also contribute to gender distinctions. In terms of high arousal, females consistently exhibit higher stimulation effectiveness in both the HAPV (38.0%) and HANV (11.1%) quadrants compared to males. However, for lower arousal, males show slightly higher stimulation effectiveness in both the LANV (2.7%) and LAPV (32.8%) quadrants compared to females.

This not only reveals gender-specific variations in emotional stimulation but also provides valuable clues for further exploring the relationship between individual emotional experiences and driving performance.

Furthermore, the ground truth for labeling driver facial expression data was derived from participants’ self-reported emotion scale data.

### Conclusion and future work

We present ViE-Take, a Vision-driven dataset for charting the Emotional landscape in Takeover of autonomous driving. ViE-Take is motivated by the conflict between the significance of emotion in takeover and the lack of specialized datasets studying the issue. It benefits both researchers from academic and engineers from industry in (a) presenting the first public dataset (to the maximum of our knowledge) to study how emotion qualitatively and quantitatively affects different aspects of takeover performance (i.e., readiness, reaction time, and quality); (b) offering 4 pretrained deep models (with full codes and parameters) for predicting takeover performance, corresponding to 4 prevailing learning strategies (2D, 2D + Timing, 2D + Timing + Attention, and 3D); and (c) providing rich emotion stimulus (i.e., clips collected from social media and movies) with manual labels, which is proved to be capable of eliciting diverse emotions.

There still exist several interesting open issues for our study. We list 2 major ones here as our future work:

1. Age. We chose participants in a narrow age range (23 to 29 years old) for easier emotion elicitation and consistent physical functionality [40]. However, it is important to extend this setting to study how emotion matters in different age ranges since different ages respond differently to emotion stimuli and perform differently in physical tasks.

2. Emotion as output. We only designed end-to-end baseline networks to predict takeover performance. However, understanding the driver’s emotions is also important for various subsequent tasks like driving intervention and emotion regulation. Thus, combining performance prediction and emotion recognition as a multi-task learning task is an interesting extension since it enriches the diversity of outputs and thus benefits downstream applications.

## Methods

### Related research

#### Emotion in driving

While the impact of emotion on manual driving has been extensively studied, its influence on takeover performance in autonomous driving has only recently garnered attention. Emotion is long recognized as an important factor affecting driving safety in manual driving [41–43]. Anger as a powerful emotion receives the widest attention as it can result in hazardous actions, including speeding and infractions of traffic regulations [44,45]. Also, it lowers the perceived safety of drivers and thus degrades their driving performance [46]. As a result, it becomes one of the most significant contributors to fatal crashes [47] (e.g., increasing the risk of a crash by 9.8 times [48]). Besides anger, other commonly experienced discrete emotions in driving like happiness, sadness, fear, and boredom (fatigue) have also been studied [46,49–51]. Their impacts on various aspects like risk perception, response, and steering have been examined.

Despite that how emotion affects manual driving has been well studied, how emotion affects takeover in autonomous driving remains unexplored until recently. Du et al. [26] presented the first empirical study in a driving simulator where participants experienced takeovers under L3 automation while watching movie clips for emotion induction. Their analysis suggests that drivers in positive valence tend to make a smaller acceleration and jerk when re-taking the control, leading to better driving quality. They also show that high arousal does not essentially lead in shorter takeover time, contrary to the observation in manual driving. Their work provides critical insights into the role of emotions in takeovers and inspires researchers to explicitly consider emotion as a major factor in predicting driver takeover performance [52].

#### Takeover performance datasets

As concluded in Table 5, there currently exists 3 public datasets on takeover performance understanding, i.e., [53–55]. Zhang et al. [53] presented a full-text dataset containing 129 takeover records retrieved from various eligible sources, including conference/journal publications, thesis, reports, posters, and presentation slides. The purpose of this dataset is to investigate how the mean takeover time varies with various experimental conditions, including the urgency of the situation, the use of a handheld device, and engagement in visual nondriving tasks. This dataset is quite useful in understanding takeover performance in reaction time but does not directly contribute to the design of performance prediction systems. Qiu et al. [54] collected a physiological signal dataset [i.e., gaze, heart rate, and galvanic skin response (GSR)] with *28* participants in simulated driving environments. They developed a prediction system for driver’s takeover intention, leveraging a 3D convolutional neural network (3D-CNN) learning on the driver physiological data as well as various driving contexts like vehicle, navigation, and weather information. Deng et al. [55] provided a similar physiological signal dataset [i.e., electroencephalography (EEG), heart rate, and GSR] with 20 participants in a driving simulator, focusing on a different metric of takeover performance, i.e., readiness.

**Table 5.** Comparison of public takeover datasets in autonomous driving. GSR, galvanic skin response; HR, heart rate; FE, facial expression; EM, eye movement; HP, head pose; BP, body posture [53-55].

Ref.	Takeover metric	Driver context		Driving context	Vision based	Setting	Emotion stimulus	Driver data	Data annotations	Participant
		Emotional	Physiological							
[53]	Takeover time	×	√	√	×	Simulator and real	×	×	×	4,556 subjects [53] (from 129 studies)
[54]	Takeover intention	×	√	√	√	Simulator	×	Gaze HR GSR	×	28 subjects
[55]	Readiness	×	√	√	×	Simulator	×	GSR HR EEG	Takeover readiness	20 subjects
ViE-Take	Readiness Reaction time Quality	√	√	√	√	Simulator	√	FE EM HP BP	Emotional statement Takeover readiness Reaction time Takeover quality	21 subjects (12M and 9F)

In summary, although emotions play a crucial role in takeover performance, there is currently no public dataset that incorporates emotions to support a comprehensive understanding of their impact on takeover performance. More importantly, such a dataset would be highly valuable for developing and pretraining an online system to predict takeover performance. Therefore, we present ViE-Take, the first Vision-driven dataset for exploring the Emotional landscape in Takeover safety, with several distinctive features, i.e., multi-source emotion elicitation, multi-modal driver data, and multi-dimensional emotion and takeover performance annotations.

### Dataset building methods

#### Multi-source emotion stimulus selection

##### Raw video–audio clips collection

Emotion elicitation is crucial for studying the correlation between drivers’ emotions and takeover performance. Therefore, selecting appropriate stimuli to elicit drivers’ emotions is important [56]. We opted for video–audio clips for emotion elicitation for the following reasons:

• Prior research has verified that watching video–audio clips reliably elicits emotional responses in drivers [57,58].

• Watching video–audio clips is one of the most common activities for drivers in the autopilot mode, as pointed out in [59].

Raw clips are selected through the following 3 sources:

Movies: We follow most prior literature and leverage movie clips for emotion elicitation (Ekman et al. [60], Gross and Levenson [61], Lisetti and Nasoz [62], and Uhrig et al. [63]). We find that the choice of movies as well as the scenes are critical in emotion elicitation since it usually requires context to understand a short movie clip. Examples such as *Mr Bean* [64] aim to deliver specific emotions, such as happiness or horror, among audiences.

Short videos from social media: Short videos are viral in social media since they fit the fast-paced lifestyle and fragmented leisure time. They are deliberately made to be easily understandable and very empathetic, making them perfect stimulus for emotion elicitation [58]. We gathered such clips from platforms like Bilibili, a well-known social media in China.

Image with background music: We also tried images with background music as stimulus, where the former comes from International Affective Picture System (IAPS) [65] and Chinese Affective Picture System (CAPS), while the latter is chosen from [66].

As shown in Table 6, we have 96 clips in total, which spread over all 4 emotional quadrants (each clip is for inducing emotions in one specific quadrant). The whole duration is over 3 h.

**Table 6.** Summary of video–audio clips stimulus collection

Raw video–audio stimulus collection	
Raw stimulus database	Movies
	Short videos from social media
	Image with background music
No. of raw stimulus	96
Stimulus duration	1–11 min
No. of rating per stimulus	6 (2 males and 4 females)
Rating values	Discrete scale of 0–1
	0 “Ineffective”
	1 “Effective”
Selected video–audio stimulus	
H, high; L, low; P, positive; N, negative; A, arousal; V, valence	
Emotion	Four quadrants based on VA model
	- HANV - HAPV
	- LANV - LAPV
HAPV	
No. of video–audio clips	32
Duration	85.1 min
Main source	Movie segment: *Tom and Jerry* Short video: bilibili
HANV	
No. of video–audio clips	19
Duration	88.77 min
Main source	Movie segment: i.e., *Spider-Man 2* Short video: bilibili (i.e., disaster)
LANV	
No. of video–audio clips	9
Duration	56.72 min
Main source	Short video: bilibili (i.e., mathematical analysis)
LAPV	
No. of video–audio clips	12
Duration	63.72 min
Main source	Movie segment
	Short video: bilibili (i.e., traveling)
Total selected stimulus	
No. of video–audio clips	71
Duration	294.31 min
Video clips format	MP4
Image Resolution	1920*1080

##### Selection process

We conducted a within-participants study to evaluate 96 different video–audio clips.

Participants. Six participants (2 males and 4 females), aged between 19 and 28 [mean (M) = 22.17 years, standard deviation (SD) = 2.67], were recruited from a college campus to take part in this study. More details are in Table 7. All participants provided signed consent forms and received a financial reimbursement of 100 RMB for their participation.

**Table 7.** Overview on the participants (rater A to F)

Rater	Age (years)	Gender	Degree	Driving experience (years)
A	23	M	Bachelor	4
B	25	F	Master	3
C	24	F	Bachelor	2
D	19	M	Master	1
E	24	F	Master	2
F	28	F	PhD	3

Materials. Subjective assessments were performed using a 2-point scale, where 0 corresponds to “Ineffective” and 1 corresponds to “Effective” for the evaluation.

Procedure. At the beginning of the experiment, participants received instructions to sit calmly and maintain stillness for a minimum of 30 s. Following this, they performed 2 simple mathematical calculation exercises designed to establish a neutral emotional state, as detailed in [67]. Subsequently, participants were exposed to a series of 96 video–audio clips, presented in a randomized order. To minimize carryover effects from the previous clip, a 1-min break time was taken between each pair of clips. The participants can take a rest anytime they want during the process. Participants were instructed to assess each clip by responding to the question: “Do you feel that the current video has successfully elicited your target emotional quadrant? Is this video effective? Please provide a rating of 0 for ineffective and 1 for effective.” Finally, 6 assessments were gathered for each video–audio clip. To identify the most effective video–audio clips, this study considered both the Pearson correlation coefficient (PCC) and the consistency correlation coefficient (CCC) results. In the 2-scale data analysis of each video–audio clip, we first computed the mean rating from 6 participants. Then, we established a gold standard for these subjective scores by leveraging the evaluator weighted estimator (EWE) fusion method for the average value, as proposed by Schuller [68]. To maintain the original 2 categories (i.e., “Ineffective” and “Effective”), we applied a simple rounding process.

Selection criteria and result. As illustrated in Table 8, we compared each rater’s score with the gold standard and analyzed the PCC and CCC between them. The results indicated a high level of consistency among raters. Therefore, we set the selection criteria of a clip as EWE =1, indicating that at least half of the raters agree on its effectiveness. Table 6 records the final selection result and offers a concise overview of the sources and the quantity of video clips chosen for each quadrant. Our chosen stimuli comprise 71 video–audio clips with a total duration of 294.31 min. The video clips are in MP4 format, and their image resolution is 1920*1080, which is suitable for participant viewing. The selected video–audio clips primarily originate from movies and social short videos.

**Table 8.** Overview on the raters’ (ID A to F) agreement: PCC, CCC of the individual raters, and EWE (mean)

Emotion	Metrics	Rater					
quadrants		A	B	C	D	E	F
HAPV	CCC	0.12	0.37	0.32	−0.08	0.27	0.06
	PCC	0.15	0.48	0.43	−0.08	0.4	0.09
HANV	CCC	0.39	0.2	0.31	0.27	0.44	0.41
	PCC	0.4	0.21	0.34	0.27	0.53	0.41
LANV	CCC	0.24	0.05	−0.14	0.28	0.42	0.75
	PCC	0.29	0.06	−0.17	0.40	0.44	0.77
LAPV	CCC	0.36	0.09	0.6	−0.02	0.27	0.56
	PCC	0.38	0.13	0.6	−0.04	0.3	0.63
Total	CCC	0.36	0.21	0.34	0.22	0.47	0.44
	PCC	0.38	0.22	0.37	0.24	0.52	0.45

#### Multi-modal driver dataset collection

##### Participants

Twenty-one participants (12 males and 9 females), with ages ranging from 23 to 29, were recruited from a college campus to take part in this study. This age range was selected to limit age-related variability in emotional responses [69–72] and ensure consistent driving abilities [40,73]. All participants submitted signed consent forms and received a financial reimbursement of 100 RMB for their participation.

##### Apparatus

**Fig. 6.**
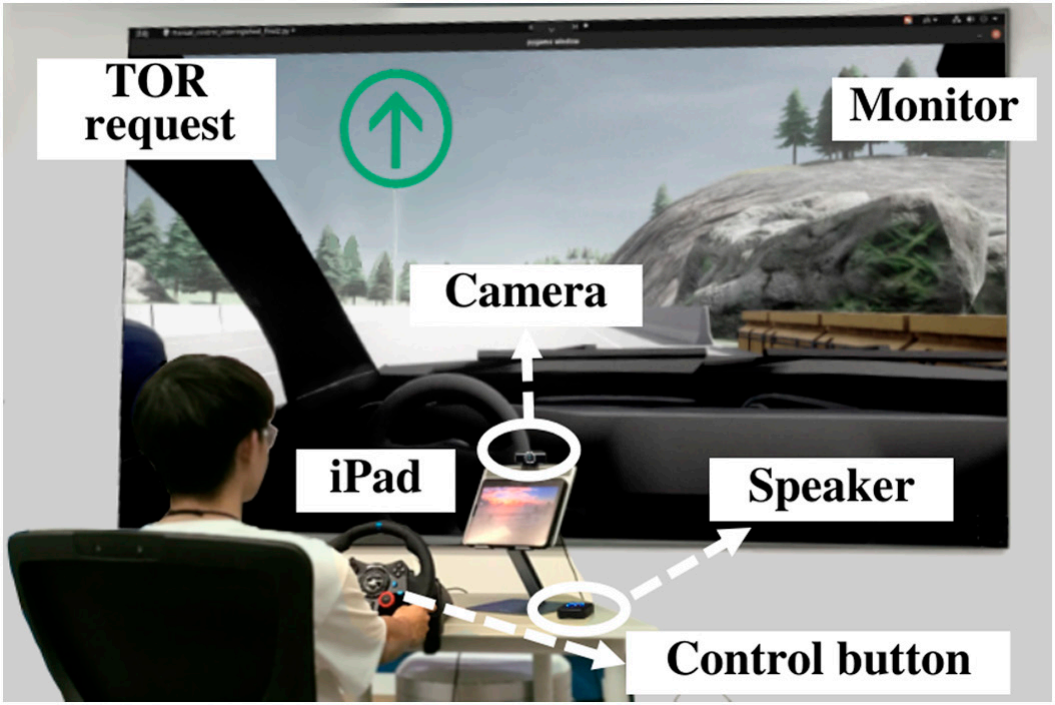
Driving simulator in the experiment.

We used the CARLA software for creating our driving simulations (we chose a simulator mainly for the safety reason as other mainstreaming works [74,75], i.e., poor takeover performance in real-world experiments could lead to car accidents, thus endangering the safety of participants [76]), as detailed in [77]. CARLA was selected for its open-source nature [78], high-fidelity simulations of driving scenarios [77], and flexibility in creating diverse driving conditions [79], making it ideal for studying both driver and automated driving tasks [5,80]. The driving scenarios were presented on a 120-inch monitor with 4K resolution (Changhong 90C7UG), as illustrated in Fig. 6. Participants were situated in the driving simulator fitted with a Logitech G29 racing wheel and corresponding floor pedals. To provide auditory and visual cues for TORs, we utilized an external audio speaker and a pop-up window [81,82]. Furthermore, an Apple iPad Pro positioned at a 30-degree angle toward the driver on an iron stand (20 cm above the dashboard) served as a supplementary visual display for watching videos. On the right side of the driver, we deployed a Rapoo C260AF web camera (30 Hz, 720p, $28.7) to capture facial expressions and behaviors throughout the experiments. The vehicle data, sampled at a rate of 30 Hz, were provided by CARLA. To further approximate real driving conditions, we have aligned the surrounding environment, in-car setup, and driving context as closely as possible with real-world scenarios.

##### Experimental setup

We followed the experimental design outlined in [26], where the vehicle is in the autopilot mode while the drive is watching videos clips (selected in the “Multi-source emotion stimulus selection” section). To prevent issues like advertisements and buffering due to unstable internet speeds, the video clips were predownloaded into the albums of the tablets. This measure was taken to ensure that participants could fully immerse themselves in the NDRTs without disruptions from external factors. For each emotional quadrant stimulus, we created a new album named after the current target emotional stimulus. For example, for the first quadrant, the new album was named “High Arousal and Positive Valence (HAPV)” and included the previously selected 32 video clips (as in the “Multi-source emotion stimulus selection” section; Table 6). For the takeover background, obstacles in the driving lane are a common occurrence for the vehicle to give back control to human drivers in real world. When a TOR is initiated, the drivers’ visual and auditory attention was engaged. Therefore, we set the TOR in an auditory warning (“Please Takeover!”) [81], accompanied by a pop-up window [82]. Furthermore, to ensure temporal alignment of both driver information and vehicle data, an additional script (in C++) was developed. This script played a crucial role in synchronizing driver-related information and vehicle data.

##### Procedure

**Fig. 7.**
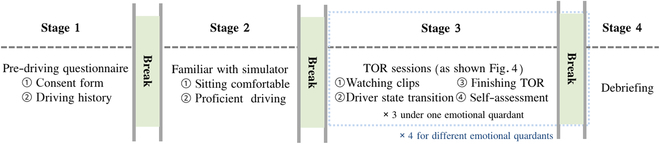
A schematic view of a completed procedure in our study, including stages 1 to 4: briefing, familiarization with the driving simulator, main driving course, and debriefing.

Experiments were conducted exclusively between 3 PM and 9 PM in the Driving Simulation Laboratory located on the university campus. Prior to this, a prerecruitment process was implemented to select eligible candidates as participants. Upon the participants’ arrival at the laboratory, an orientation session was conducted, outlining the experiment’s purpose and the procedural steps to be followed (see Fig. 7).

Stage 1: Predriving questionnaire. Prior to the experiment, the participant completed a consent form and a personal driving history questionnaire.

Stage 2: Familiar with simulator. Participants went through a 20-min training session to learn how to operate the simulator, including manual driving and answering TORs. Then, they were asked to learn efficient use of the iPad for emotional stimulation. They can freely select video–audio clips by swiping left or right to ensure that the target emotions can be successfully elicited.

**Fig. 8.**
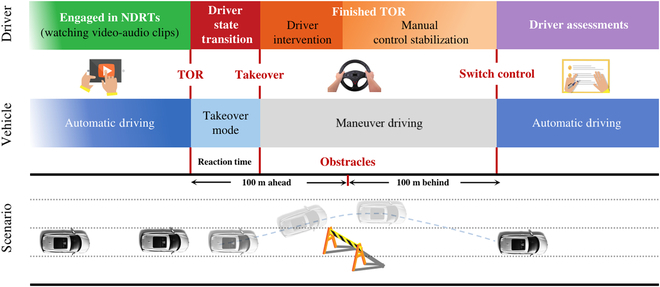
The takeover timeline, including automated driving, encountering a TOR, resuming manual driving to avoid obstacles, and back to automated driving. The deep black car indicates automated driving, while the light black car means manual driving.

Stage 3: TOR sessions (as shown in Fig. 8), which consists of the following 4 parts:

• Engaging in NDRTs (watching video–audio clips). While the autonomous vehicle travels at a speed of 60 km/h, participants select the corresponding emotion album on the Apple iPad Pro to watch videos, ensuring that they remain within a specific emotional quadrant. Participants focus on the video clips until a TOR appears. This process lasts approximately 3 min.

• Driver state transition. The TOR is set at a distance of about 100 m ahead of the obstacles. During the experiment, TOR is initiated through both auditory prompts from the speaker and pop-up window in the screen notifications, reminding the driver to take control. At this point, the driver can regain control of the vehicle by pressing a TOR button on the steering wheel.

• Finishing TOR. The vehicle is now in the maneuver mode. Participants engage in actions such as braking and steering to avoid the obstacles. Afterward, they drive the vehicle back to the second lane until they are 100 m behind the obstacle. Then, they press the same button to resume automatic driving.

• Driver assessments. With the vehicle in autonomous driving mode, indicating the completion of the entire TOR process, participants recall their true emotions during the viewing of video–audio clips and complete the SAM questionnaire. Simultaneously, they label their readiness for takeover. The experiment observer also documents the takeover quality.

Each participant was required to complete 3 TORs (stage 3) in one experiment. To minimize carryover effects from the previous emotion elicitation and mitigate driver fatigue, a 3-min break time was implemented between each pair of emotional quadrants. Additionally, a 4×4 Latin squares design [83] was employed to balance the order of inducing the 4 different emotional quadrants across participants, minimizing the potential ordering effect. This method ensures that participants would not be affected by residual emotions from previous videos [84].

Stage 4: Debriefing. After the end of the driving, participants received a debriefing sheet and 100 RMB for their participation.

#### Dataset labeling, processing, and cleaning

##### Data labeling

During the experiment, after each TOR following emotional elicitation, participants provided self-labels for their emotions and takeover readiness. Observers also label takeover performance based on the driving situation after takeover. The details are listed as follows.

Emotion labeling. After the emotion elicitation, participants were instructed to complete the SAM to assess their emotional states. The scale includes 2 dimensions: arousal and valence, each ranging from 1 to 9. Arousal is rated on a scale where 9 indicates “High” and 1 indicates “Low”, while valence is evaluated on a scale where 9 corresponds to “Positive” and 1 corresponds to “Negative”.

Additionally, we normalize the original 1 to 9 scale to [−1, 1] using the maximum–minimum method:

$$X_{\text{normalized}}=\frac{X-X_{min}}{X_{max}-X_{min}}$$ (3)

where X represents the original variable, $X_{\min}$ denotes minimum values, $X_{\max}$ is maximum values, and $X_{\text{normalized}}$ is the normalized result, respectively. This transformation is applied to both valence and arousal values for enhanced comparability (Eq. 3).

Takeover performance labeling. Regarding the labeling of takeover performance indicators, detailed information can be obtained from Table 2.

• Readiness was assessed through subjective indicators, evaluated subjectively by the participants themselves, using a scale ranging from 0 to 1. After each takeover event, participants indicated their readiness level, where 0 represented “Not ready” and 1 indicated “Ready”.

• Reaction time was objectively measured on a scale from 0 to 2 using indicators from the simulator, following a normal distribution with a μ (mean) of 1.66 and a σ (variance) of 0.44 (as mentioned in the “Takeover performance indicators” section). The labeled categories are as follows: 0 for “short reaction time” (0 to 1.22 s), 1 for “medium reaction time” (1.22 to 2.10 s), and otherwise 2 for “long reaction time”.

• Takeover quality subjective labels were assigned by 4 raters (A to D) recruited from the college campus. Additionally, this assessment was complemented by objective labels encompassing acceleration, jerk, brake, steering, and ${\mathrm{TTC}}_{\min}$. During the process, raters took a 10-min break every 30 min to avoid fatigue annotation. After completing the annotation, we establish a gold standard for these 4 subjective scores by leveraging the EWE [68] fusion for the average value and performing a simple rounding to maintain the original 5 categories. Subsequently, we compare each rater’s score with the gold standard and analyze the PCC, CCC, kappa correlation coefficient, and weighted kappa correlation coefficient between them, as shown in Table 9. The results show a high level of consistency among raters, indicating the effectiveness of the labeling.

**Table 9.** Overview on the raters’ (ID A to D) agreement: PCC, CCC, kappa and weighted kappa of the individual raters and EWE (mean)

ID	PCC	CCC	Kappa	Weighted kappa
A	0.913	0.907	0.796	0.851
B	0.944	0.940	0.874	0.906
C	0.942	0.937	0.862	0.899
D	0.952	0.950	0.894	0.921

##### Data processing

Multi-modality data streams extraction. The recorded driving video is analyzed to extract multi-modal data, including face, head pose, eye movements, and body postures. Utilizing diverse information types enriches the data resources available for downstream takeover performance prediction tasks.

**Fig. 9.**
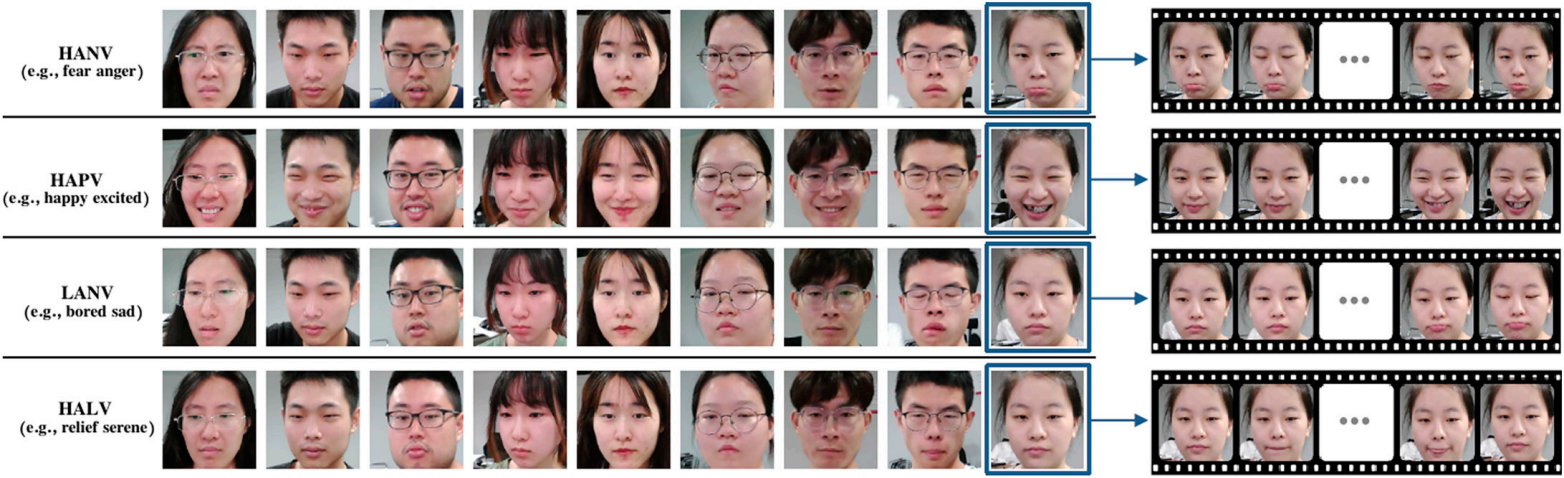
Samples of facial expressions of 4 emotional quadrants in the dataset.

Face constitutes a primary means of emotional expression [85–87]. Drivers engaged in NDRTs may experience a range of emotions when facing TORs. To effectively capture these emotional nuances, we extract facial expression features from the video data using the multi-task convolutional neural network (MTCNN [88]), with a sampling frequency set to 30 frames per second. As a result, the output of MTCNN is (30×10, 256×256×3). Several examples of dataset images are depicted in Fig. 9, illustrating the diversity of drivers’ facial expressions corresponding to different emotions.

Studies have demonstrated that eye movement and head pose are strongly associated with driving attention [89,90]. In particular, the driver’s focus, which can be assessed through their eye movement and head pose, clearly differs between distracted and nondistracted situations. The OpenFace 2.0 toolbox, as detailed in [91], has demonstrated effectiveness in extracting eye movement and head pose from video images. Hence, we utilize this toolbox for preprocessing the video data to acquire eye movement and head pose features from each frame. The specific features of eye movement extracted by OpenFace 2.0 include the direction of left eye gaze, right eye gaze, angle of both eyes, and coordinates of key points in the eye area. The total dimensionality of these eye movement features is (10×30, 288). In addition, the extracted features of the head pose include the position of the head relative to the camera and the rotation angles *X*, *Y*, and *Z* of the head. The total dimension of these head pose features is (10 × 30, 6).

Body posture can serve as a direct indicator of driving attention. By capturing the posture changes of the driver’s upper body, various information attention-related tasks can be realized, e.g., fatigue detection [92]. We use the pretrained Openpose model (see [93]) to detect limbs in frame images, providing 12 point coordinates specifically for the upper limb region in each frame.

Vehicle data like speed, throttle pedal angle, brake pedal angle, and steering wheel angle are captured at a sampling rate of 30 Hz.

The input streams are chosen through uniform temporal position sampling from synchronized video clips and vehicle data, resulting in a 15-frame sample for face, head pose, eye movement, and body posture data. Detailed configurations for the input data streams across different modalities in each sample are presented in Table 1.

Data cleaning. Data cleaning is a crucial step to maintain the quality and integrity of acquired data. This includes removing any outliers, addressing missing values, and handling noise in the recorded driving data.

**Fig. 10.**
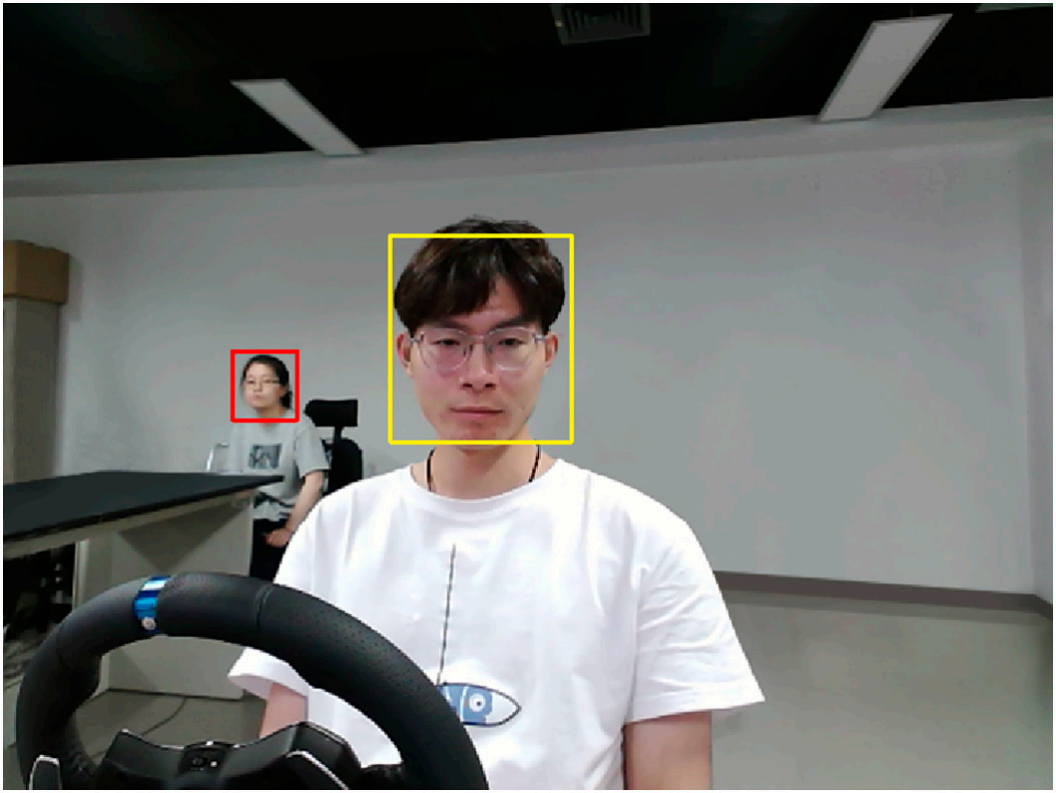
An example of wrong recognition where MTCNN failed.

We employ MTCNN for face detection; however, it may introduce noisy data that contain irrelevant faces (e.g., observers, or even faces printed on the clothes). For instance, as shown in Fig. 10, MTCNN erroneously detects the face of an experimental observer seated behind participants (red boxes highlighting), instead of accurately extracting the face of the participant (yellow boxes indicating). Therefore, we carefully manually inspect the dataset, resizing the corresponding original images. For instance, we crop images and then re-input the data, ensuring that MTCNN accurately recognizes the participant’s facial images. If misidentification persists, we discard such samples.

Here, we present examples of different participants in different emotion quadrants in Fig. 9, which indicates that our carefully selected emotion stimulus works well in practice by fulling evoking diverse emotions with explicit facial expressions. More analysis about emotion elicitation is given in the “Emotion stimulation” section.

## Data Availability

The project is available on https://insightfuleyes.github.io/ViE-Take/main.html. The dataset for [53] is available here: https://data.4tu.nl/articles/_/12704915/1; the dataset for [54] is available here: https://usa.honda-ri.com/hdbd; and the dataset for [55] is available here: https://deepblue.lib.umich.edu/data/concern/data_sets/6682x4804.
